# Nurses’ challenges and successes in caring for Syrian refugees: A qualitative phenomenological study

**DOI:** 10.1097/MD.0000000000050409

**Published:** 2026-08-28

**Authors:** Bircan Kara, Sercan Kara

**Affiliations:** a Hatay Mustafa Kemal University Hospital, Hatay, Türkiye; b Department Fundamentals of Nursing, Faculty of Nursing, Ankara University, Ankara University Graduate School Health Sciences, Ankara, Türkiye.

**Keywords:** communication barriers, cultural competence, refugee healthcare, support systems

## Abstract

Türkiye hosts the world’s largest Syrian refugee population. This places major demands on healthcare staff. Nurses in border areas such as Hatay face specific challenges. They must provide care that respects cultural differences. This study explored the lived experiences of Turkish nurses caring for Syrian refugees. It focused on care challenges and successful adaptation strategies. This qualitative phenomenological study used Colaizzi’s 7-step method. It included 16 nurses in Hatay, Türkiye. All participants provided direct care to Syrian refugees. Data were collected through face-to-face, semi-structured interviews. Each interview lasted 30 to 40 minutes. Trustworthiness was supported by member checking, peer debriefing, and an audit trail. Four main themes emerged from the analysis. The first theme covered challenges in working with refugees. Nurses described different health beliefs and language barriers. These barriers made it difficult to obtain accurate health histories. The second theme concerned difficulty in understanding health needs. It included gender-sensitive care, signs of trauma, and symptoms lost in translation. The third theme covered barriers to healthcare access. These included fear of procedures, long registration steps, and a lack of trained interpreters. The fourth theme focused on support and successful strategies. Nurses used mobile translation applications, help from coworkers, professional networks, and emotional regulation strategies. These methods offered immediate help, but sustainable support systems were limited. Nurses caring for Syrian refugees showed resilience and developed informal coping strategies. However, institutional support remained limited. Training programs should be revised. Staff also need reliable human and digital resources. These resources may reduce cultural and language barriers in refugee care.

**Key Points**

- Nurses caring for Syrian refugees face major language and cultural barriers. They also manage complex health needs and high emotional stress.
- This study identifies care challenges and successful strategies. It also shows the need for cultural sensitivity and clear communication.
- Emotional support and targeted training may improve patient outcomes and nurses’ well-being.
- The findings may guide culturally sensitive care protocols. They may also support policies that protect the mental health of healthcare staff.
- The findings may inform policy and practice by showing factors that reduce barriers in refugee care.

## 1. Introduction

Recent world events have increased movement across borders and the number of refugees.^[1]^ This trend may continue. Türkiye hosts about 3.7 million registered Syrian refugees under temporary protection. This is the largest Syrian refugee population in the world.^[2]^ Hatay is an important setting, but few studies have focused on it. It is Türkiye’s southernmost province and shares a 120-kilometer border with Syria. Hatay also has one of the highest refugee rates in the country. Registered Syrians make up about 15% to 20% of the population.^[3,4]^ Since 2011, healthcare centers in Hatay have treated many more patients. This has placed added strain on a public health system with limited resources.^[5,6]^ No previous phenomenological study has examined nurses’ experiences in this main reception area. Earlier studies show language and cultural barriers in host healthcare systems.^[7,8]^ Nurses also report greater workloads and emotional strain when caring for patients from other countries.^[9,10]^ Yet little is known about how nurses cope in areas with a very high refugee load. It is also unclear which strategies they develop and which forms of support work over time. This study examines barriers and successful strategies from the nurses’ perspective in Hatay. Refugees may face economic hardship and barriers to basic services, including education and health care. Health care can be costly and difficult to access. Refugees may also struggle to adapt to a new culture. Language, health beliefs, cultural differences, and social norms can limit care.^[11]^ People may receive poorer care when their language or culture differs from that of the host country. They may also face health inequalities and major barriers to access.^[12,13]^ Care for these vulnerable groups must be safe, fair, and humane.

Humane care requires respect for each person’s culture. Healthcare staff need to understand attitudes, behaviors, values, body language, and forms of expression.^[14]^ Nurses have close and frequent contact with patients. They are expected to provide this type of care.^[15]^ It is therefore important to understand how nurses care for refugees from other cultures. It is also important to identify the barriers that affect care.^[16]^

Nurses in Türkiye have cared for Syrian refugees since the war began. Their challenges and successful strategies can guide future care. This knowledge may help nurses respond to new refugee movements. It may also help decision-makers take practical steps. These steps can improve access and the quality of care. Health care is a basic human right.

This study examined the challenges, barriers, and successful strategies reported by Turkish nurses caring for Syrian refugees.

The study addressed the following research questions:

**Question (Q)1**: What challenges do Turkish nurses face when interacting with Syrian refugees?
Q2: What barriers do Turkish nurses face while providing care to Syrian refugee patients?
Q3: What successful strategies do Turkish nurses use while providing care to Syrian refugee patients?

## 2. Methods

### 2.1. Study design

This study used a descriptive phenomenological approach and followed Colaizzi’s 7-step method. This approach is based on Husserl’s philosophy. It aims to describe the core meaning of human experience through a close review of participant accounts.^[17]^ The method was suitable for studying nurses who cared for Syrian refugees. It allowed the researchers to examine professional challenges, emotional responses, and adaptation strategies. It also provided a clear process for studying experiences that are hard to measure. This helped preserve the nurses’ own voices.

Colaizzi’s method was chosen because it provides clear steps. These steps were: reading all data; identifying key statements; stating their meanings; grouping meanings into themes; writing a full description; identifying the core structure; and checking the findings with participants.^[18,19]^

### 2.2. Research design and participants

The sample included 16 nurses from a public hospital in Hatay, Türkiye. All provided direct care to Syrian refugees. Purposeful sampling was used to select participants with rich experience.^[20]^ Nurses could take part if they: had at least 1 year of nursing experience; provided direct care to Syrian refugees; and agreed to participate.

**Figure 1.**
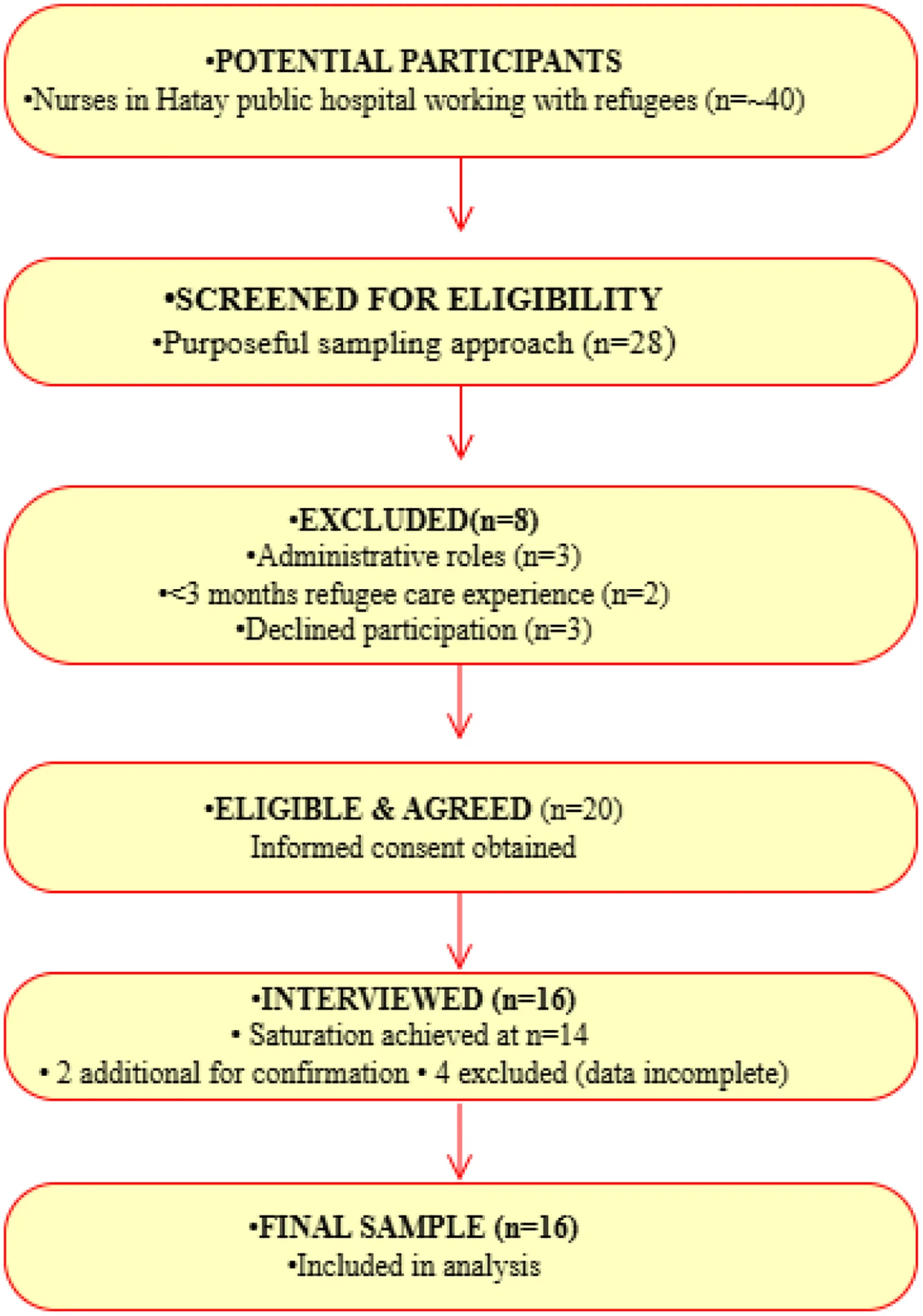
Data collection process flowchart.

Nurses were excluded if they: held administrative roles without direct patient care; had <3 months of experience in refugee care; or had joined a similar study in the past 6 months. Sample size was based on data saturation. This was defined as 3 interviews in a row with no new codes or themes. Data were collected and reviewed at the same time. This helped ensure that the themes reflected shared experiences. Saturation was reached at interview 14. Two further interviews produced no new data. The final sample included 16 nurses (Fig. 1).

### 2.3. Data collection

The researchers developed a semi-structured interview guide based on the study questions. It covered 4 areas: professional challenges; language and culture; hospital and system barriers; and adaptation strategies and sources of support. Four open-ended questions explored care challenges and successful strategies. The questions were based on previous studies.^[13,21–23]^ They were tested with 2 nurses who were not included in the final sample. Sample questions were: “Can you describe a typical day when caring for Syrian refugee patients?”; “What problems do you face when communicating with refugee patients?”; and “What strategies have helped you manage these problems?” An expert in qualitative methods reviewed each question. The expert confirmed that the questions were open-ended and did not lead participants.

The interviews were held face-to-face in private hospital rooms. Each interview lasted 30 to 40 minutes. With consent, all interviews were audio-recorded. The first author transcribed each recording word for word. The transcripts were checked against the recordings for accuracy. Field notes were written immediately after each interview. The study followed the COREQ reporting guide.

The interview guide contained 4 open-ended questions. These questions encouraged detailed responses without leading participants toward fixed answers. They moved from broad professional challenges to specific care barriers and support systems. The domains and questions were as follows:

Professional Challenges Research Question 1 (RQ1): “What are the biggest challenges in working with Syrian refugees?”

Health Needs Assessment (RQ2): “What difficulties do you encounter in understanding the health needs of refugees?”

Support Resources (RQ3): “Which support resources are helping you?”

Systemic Barriers (RQ1 & RQ2): “What are the biggest obstacles refugees face in accessing healthcare services?”

### 2.4. Data analysis

Data were analyzed with Colaizzi’s 7-step method.^[21,24,25]^ The steps were: reading the transcripts several times; identifying key statements; stating their meanings; grouping meanings into themes; writing a full description; identifying the core structure; and checking the findings with participants.

MAXQDA 11 was used to manage the qualitative data. It supported clear data storage and made the coding process easy to follow. It also allowed themes to be compared across participants. Two researchers independently coded 20% of the data. They agreed on 68 of 80 codes (85%). All other coding differences were resolved through discussion.^[25,26]^ A full record of decisions, theme changes, and coding changes was kept. This supported the dependability of the analysis.

#### 2.4.1. Trustworthiness

The criteria of Lincoln and Guba were used to support trustworthiness (Table 1).^[27–29]^ Peer debriefing was conducted by Associate Professor SŞ, an expert in qualitative research. The expert independently reviewed the coding and theme development.

**Table 1:** Criteria of Lincoln and Guba’ s.

Criterion	Strategy
Credibility	Member checking with 4 participants; peer debriefing, prolonged engagement (6 months)
Transferability	Thick description of context; detailed documentation of setting and participants
Dependability	Audit trail; stepwise replication, inter-coder agreement (85%)
Confirmability	Reflexive journal maintained by first author, bracketing; independent verification

Member checking: After the analysis, 4 nurses (25%) reviewed their interview summaries and the identified themes. This confirmed that the findings reflected their experiences.^[25,30]^

Thick description: Detailed information is provided about refugee care in Hatay, the hospital, and the participants. Readers can use these details to judge whether the findings apply to similar settings.

Audit trail: Complete records of analytic decisions, coding changes, and theme development were maintained.

### 2.5. Ethics committee approval

Ethics approval was obtained before the study. Approval was granted by the Non-Interventional Ethics Committee of Hatay Mustafa Kemal University. The decision number was 63, dated January 8, 2025. The hospital also approved the study on December 17, 2024 (No. 453977). Permission was obtained from the unit nurse managers. Participants were told the study’s aim and how the results would be used. Both verbal and written informed consent were obtained. The study followed the Declaration of Helsinki, Good Clinical Practice, and standards for reporting qualitative research.

### 2.6. Bias and reflexivity

#### 2.6.1. Researcher’s position

The first author is a lecturer at the hospital where the data were collected. This role may have affected the study. Participants may have softened their answers because they knew the researcher. The researcher’s past work with Syrian refugees may also have shaped the questions and the interpretation of the data.

#### 2.6.2. Reflection strategies

The strategies used to address these concerns are presented in Table 2.

**Table 2:** Used reflection strategies.

Strategy	Application
Bracketing	Explicit Acknowledgments and suspension of prior assumptions about refugee care during interviews
Reflexive journaling	Detailed documentation of emotional responses, assumptions, and analytical decisions maintained throughout data collection and analysis
Independent verification	Second author (external to hospital) independently reviewed 20% of coding; inter-coder agreement 85%
Peer debriefing	Qualitative research expert reviewed coding process and theme development.
Transparency	Full disclosure of researcher role to participants; assurance of confidentiality and voluntary participation
Prolonged engagement	6-month data collection period allowing trust-building and observation of consistent patterns

#### 2.6.3. Steps to reduce bias

Nurses were selected from different units. Open-ended questions were used and did not lead participants. Two researchers reviewed the data independently. A full record of all analytic decisions was also kept.

## 3. Results

### 3.1. Descriptive results

A total of 16 nurses participated. Nine were women (56.2%), and 7 were men (43.8%). Their mean age was 32.2 ± 5.0 years (range, 26–44 years). They had a mean of 12.4 ± 6.6 years of nursing experience (range, 5–28 years). Twelve participants held a bachelor’s degree (75.0%), and 4 held a master’s degree (25.0%). Eight worked in surgical units (50.0%), and 8 worked in internal medicine units (50.0%). Nine participants (56.2%) reported no foreign language skills. Seven (43.8%) spoke at least 1 foreign language. Three spoke English only, 1 spoke Arabic only, and 3 spoke both English and Arabic. Table 3 presents the full sociodemographic data.

**Table 3:** Sociodemographic characteristics of nurses.

	Age	Gender	Service worked on	Yr of study	Education	Foreign language
P1	27	Male	Internal	5	Bachelor’s degree	English-Arabic
P2	28	Female	Surgical	6	Bachelor’s degree	None
P3	33	Male	Surgical	9	Bachelor’s degree	English
P4	26	Female	Internal	13	Bachelor’s degree	None
P5	27	Male	Internal	12	Master’s degree	English
P6	29	Female	Internal	14	Bachelor’s degree	None
P7	32	Male	Surgical	22	Master’s degree	English-Arabic
P8	30	Female	Surgical	8	Bachelor’s degree	None
P9	44	Female	Surgical	28	Master’s degree	None
P10	33	Male	Internal medicine	14	Bachelor’s degree	English
P11	40	Female	Surgical	22	Bachelor’s degree	None
P12	33	Female	Internal medicine	13	Bachelor’s degree	None
P13	29	Male	Surgical	8	Bachelor’s degree	Arabic
P14	37	Female	Surgical	9	Bachelor’s degree	None
P15	32	Female	Internal medicine	6	Bachelor’s degree	None
P16	36	Male	Internal medicine	9	Master’s degree	English-Arabic

P = Participant.

### 3.2. Thematic analysis results

**Figure 2.**
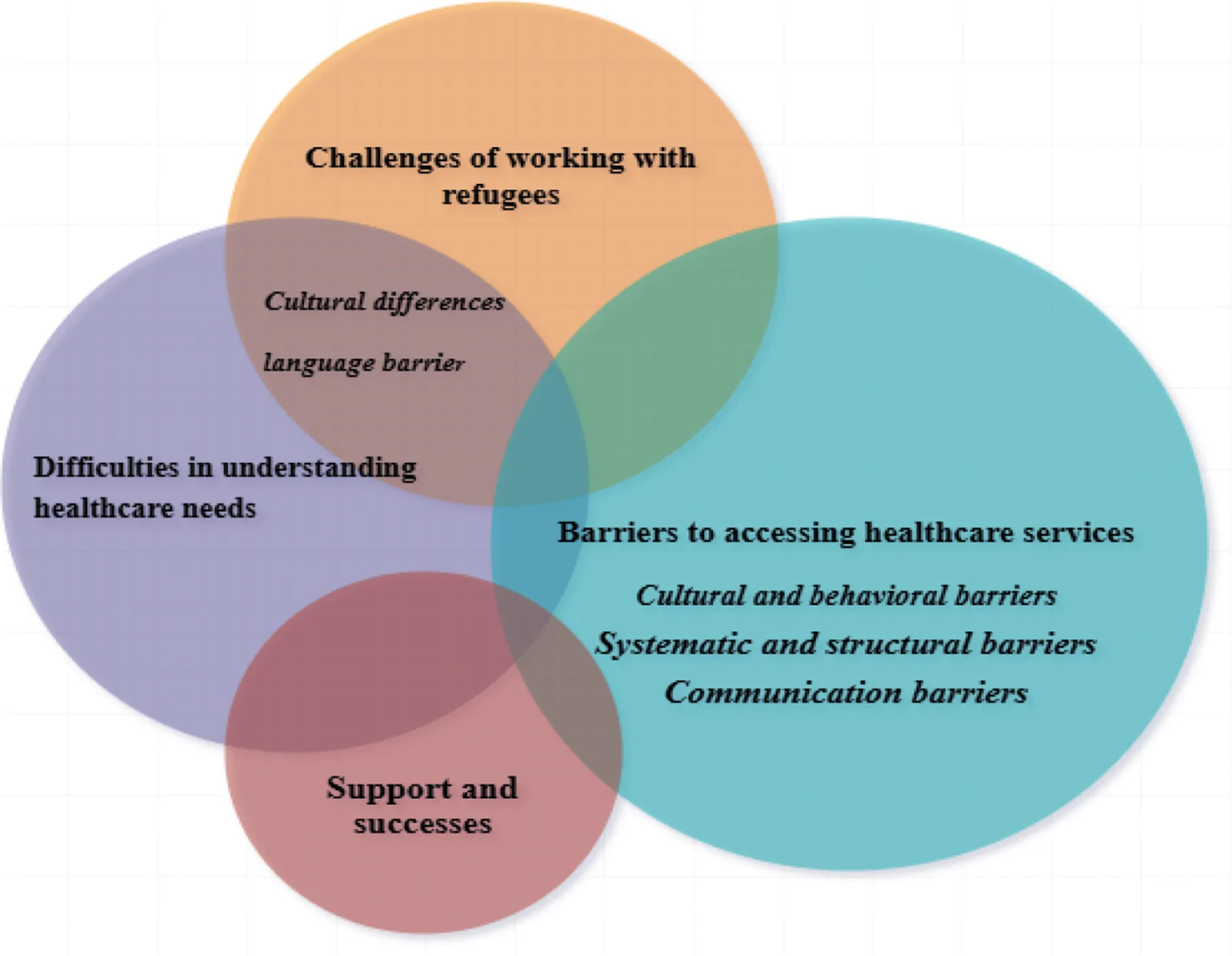
Venn diagram showing the intersections of main themes and subthemes.

The analysis identified 4 themes and 7 subthemes. Table 4 presents them in the code-matrix browser. The findings are reported by theme (Table 4 and Figure 2).

**Table 4:** Themes, subthemes and codes of nurses’ experiences (N = 15).

Theme	Subthemes	Codes	Frequency (n)
1. Communication and cultural barriers	Linguistic discordance	Language barriers, misinterpretation of symptoms	15
	Cultural misalignment	Culture shock, gender-sensitive care issues, religious norms	12
	Information gap	Incomplete medical history, Lack of previous health records	10
2. Structural and operational challenges	Systemic pressure	Increased patient volume, triage difficulties, long waiting times	14
	Resource constraints	Inadequate physical space, Shortage of specialized equipment	9
	Workforce strain	Staff shortages, high turnover in border regions	7
3. Emotional burden and compassion fatigue	Psychological impact	Feelings of helplessness, secondary traumatic stress, empathy fatigue	11
	Professional frustration	Exhaustion due to repetitive barriers, Moral distress	13
	Coping deficits	Lack of institutional psychological support, burnout risk	8
4. Support and successes	Communication support	Interpreter support, technology-based translation programs	12
		Arabic-speaking healthcare personnel, Turkish-speaking family members	11
	Professional collaboration	Assistance from hospital staff for gender-based care	1
	Institutional progress*	Recruitment of permanent interpreters, refugee support desk	6

* Institutional progress codes reflect changes implemented following the communication of study findings to the administration.

#### 3.2.1. Challenges of working with refugees

**Figure 3.**
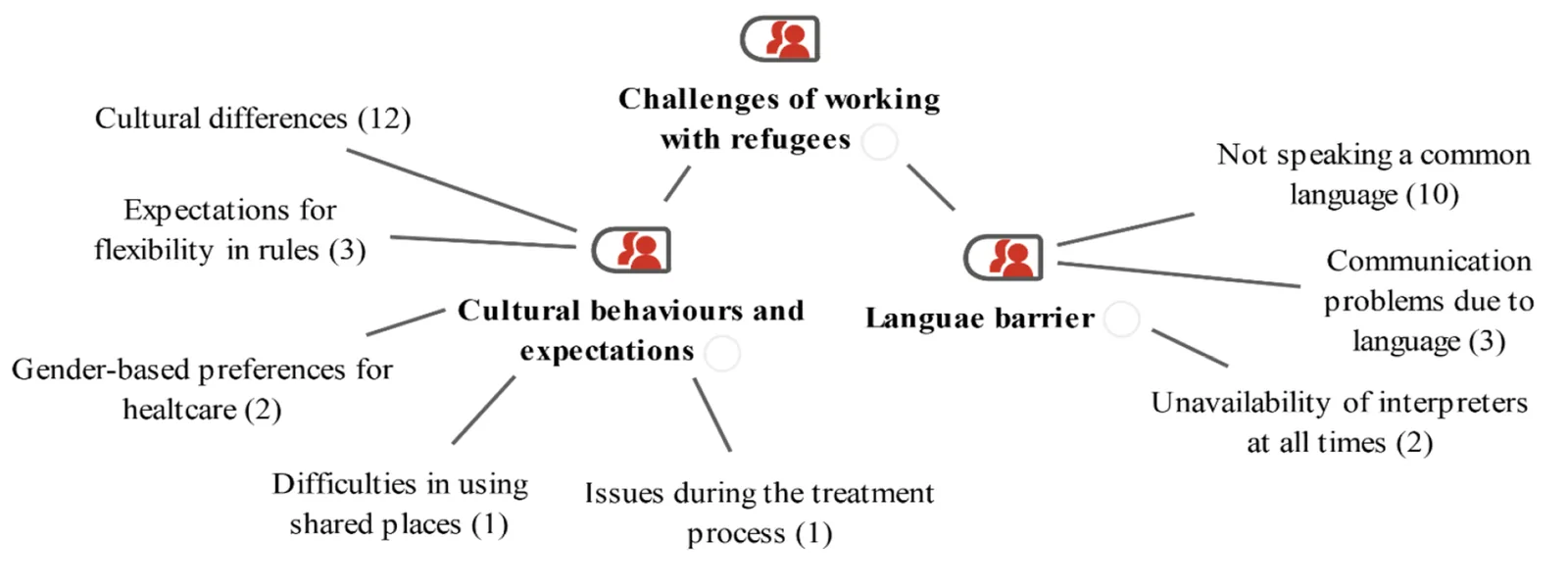
Subthemes, codes, and frequencies related to the theme “Challenges of working with refugees.”

Figure 3 presents the 2 subthemes, codes, and frequencies for the theme “Challenges of working with refugees.”

Participants were asked, “Which challenges do you face most when working with Syrian refugees?” Most nurses described cultural challenges. Refugees often arrived in large groups. They wanted more than 1 person to stay with the patient and asked staff to relax visitor rules. Some patients had difficulty following treatment rules. For example, some were unfamiliar with scheduled medication times. Nurses said that repeated requests for special rules made care harder. A few nurses also reported problems when women did not want care from male staff. This was most clear during procedures that required privacy. One nurse said that some refugees did not want to share rooms with local patients. This made bed and room planning more difficult. Another nurse said that some patients left before treatment ended. This interrupted treatment and placed their health at risk.

Language was another major challenge. Many nurses said that the lack of a shared language made communication and care planning difficult. It also prevented nurses from fully understanding patient needs. Some patients could not explain their needs, and nurses could not clearly explain care or treatment. A few nurses said that the lack of an interpreter was most difficult at night. At these times, communication could become almost impossible. This could delay or interrupt care.

**Participant 13 (P13**): *“I don’t speak Arabic, and they don’t speak Turkish; this issue is very difficult for us. When we cannot communicate with the patient, we have difficulty even in planning the patient’s care. Sometimes they ask us to bend the rules, and they are very insistent on that, which also challenges us.”*

P6: *“We have cultural difficulties. Eating and drinking, clothing, and family structures are different. For example, they try to enter crowded areas where visitors are not allowed, and we have to constantly warn them about this. On the other hand, sometimes we cannot leave the rooms with the local people. There are some problems.”*

P8: *“General language, culture, lifestyle, and behavior are different. We have difficulties with these issues. We have difficulty understanding and communicating with them. Sometimes they want to go out or leave the hospital without permission or before their treatment is completed. This is also difficult for us.”*

#### 3.2.2. Difficulties in understanding healthcare needs

**Figure 4.**
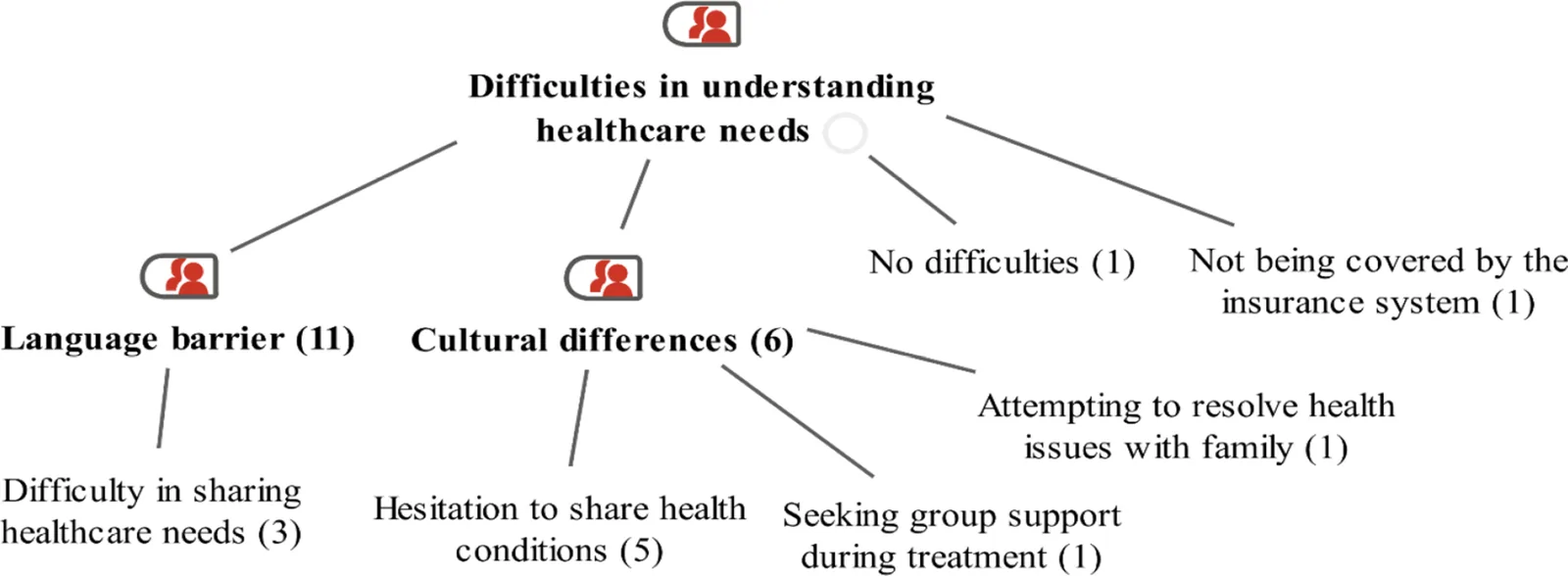
Subthemes, codes, and frequencies related to the theme “Difficulties in understanding healthcare needs.”

Figure 4 presents the 2 subthemes, codes, and frequencies for the theme “Difficulties in understanding healthcare needs.”

Participants were asked, “What problems do you have in understanding refugees’ health needs?” Most nurses identified language as a main challenge. Without a shared language, nurses could not communicate clearly or understand all patient needs. Some patients could not explain their health problems. This made it difficult to share information and plan care. Nurses also described cultural challenges. Some refugees did not understand the health system or had different expectations of care. Many nurses said that some patients felt shame when discussing illness. Some hid pain because they viewed it as a sign of weakness. This made health needs harder to identify. One nurse described gaps in health insurance coverage as a major barrier. Another said that large family groups made care planning harder. Some refugees first tried to manage health problems within the family. This could delay professional care. One nurse who spoke Arabic and knew the culture reported no such problem.

P7: *“…As I said, I speak Arabic, and I do not have difficulties because I know their culture…”*

P1: *“…Sometimes they try to explain, for example, their pain, but I cannot understand many things because I cannot communicate directly. Unfortunately, it is impossible to meet their needs completely; they do not have an insurance system, and it becomes impossible to cover some procedures.”*

P3: *“We have difficulty in understanding the needs because we do not speak the language. Unfortunately, these difficulties are also reflected in the patient’s treatment process. For example, if the patient is vomiting, we ask them to tell us. In this regard, due to their culture, they inform their relatives about such needs and try to solve them with their relatives. This also challenges us because we cannot intervene quickly and on time.”*

P13: *“…Sometimes their cultural behavior prevents us from understanding their needs…. Another thing is that they hesitate to tell you about pain.”*

#### 3.2.3. Barriers to accessing healthcare services

**Figure 5.**
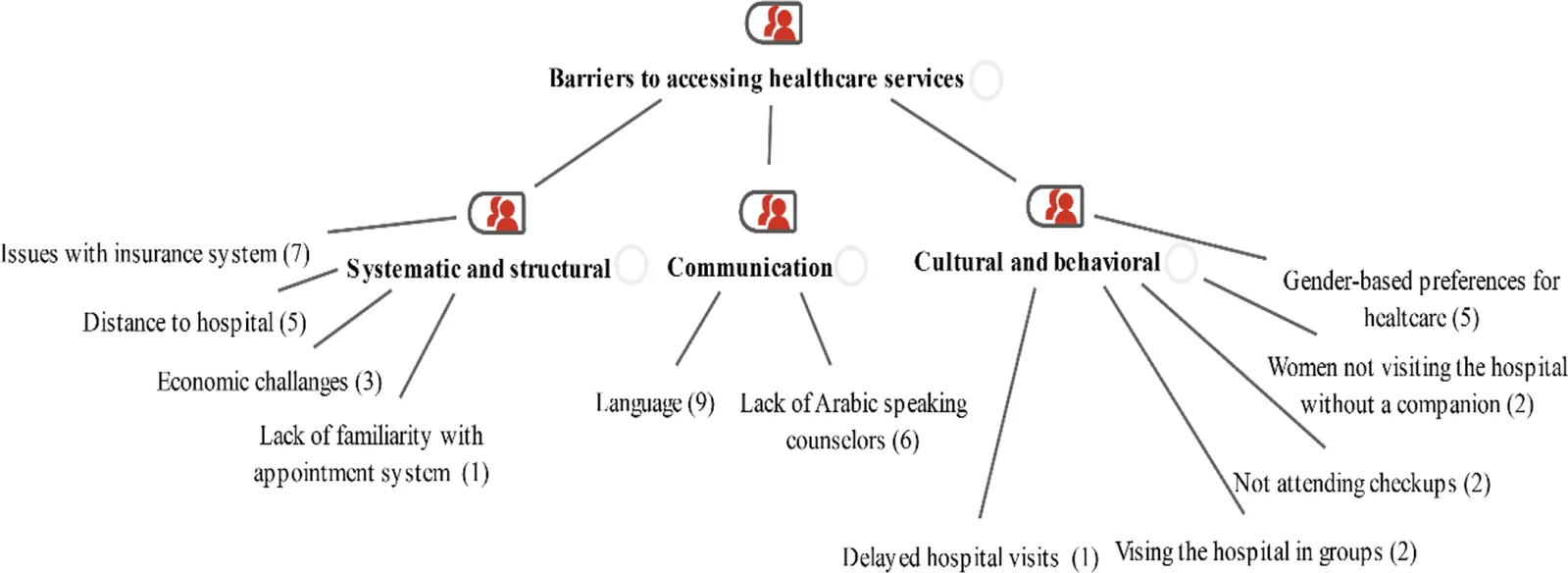
Subthemes, codes, and frequencies related to the theme “Barriers to accessing healthcare services.”

Figure 5 presents the 3 subthemes, codes, and frequencies for the theme “Barriers to accessing healthcare services.”

Participants were asked, “What barriers do refugees face when accessing health services?” The findings formed 3 subthemes: cultural and behavioral barriers, systemic and structural barriers, and communication barriers.

Several nurses described gender preferences as a cultural and behavioral barrier. Some women did not want care from male staff. A few nurses said that crowded patient rooms made care and treatment difficult. Some women did not visit the hospital without a spouse or another companion. This also limited access. Missed follow-up visits and delayed hospital visits were other key problems.

Most nurses said that refugees had problems obtaining medicines and supplies. This could occur even when they had insurance coverage. Several nurses said that distance from the hospital also limited access. Some nurses described economic barriers. One nurse said that many refugees did not know how the appointment system worked. Language was the most common communication barrier. Most nurses said that not speaking Turkish made access and care difficult. Several said that refugees did not know which clinic to visit or where to go. A lack of Arabic-speaking guides made this problem worse. The problem was greatest during the first hospital visit.

P6: “*Especially the language barrier comes to the fore. Even if they try to access health services when they do not speak the language, there are problems in this context. There are also cultural problems, of course, for example, sometimes they do not allow male nurses to take care of female patients or male doctors to take care of female patients, which is a big obstacle in accessing health services.”*

P1: *“When we are caring for or treating the patient, they are crowded, and even if you take them out of the room, they are crowded in the patient’s room while one person should stay with them. This is quite challenging in care and treatment processes.”*

P4: *“…They do not know where to go when they come to the hospital, they do not know which branch they will be examined. They definitely need counseling. Sometimes in discharge trainings, you tell them the date of the check-up, and there may be a situation that we cannot come. Naturally, the patient’s treatment process is prolonged. This time, their access to hospitals and health services is highly jeopardized [...] a woman patient cannot come to the hospital alone without her husband or companion.”*

P5: *“When they come to receive services, they don’t know where to get them.” They don’t know where to go or which departments to visit, and this is due to their lack of language skills […] the waiting times increase significantly because they do not know how to make an appointment.”*

#### 3.2.4. Support and successes

**Figure 6.**
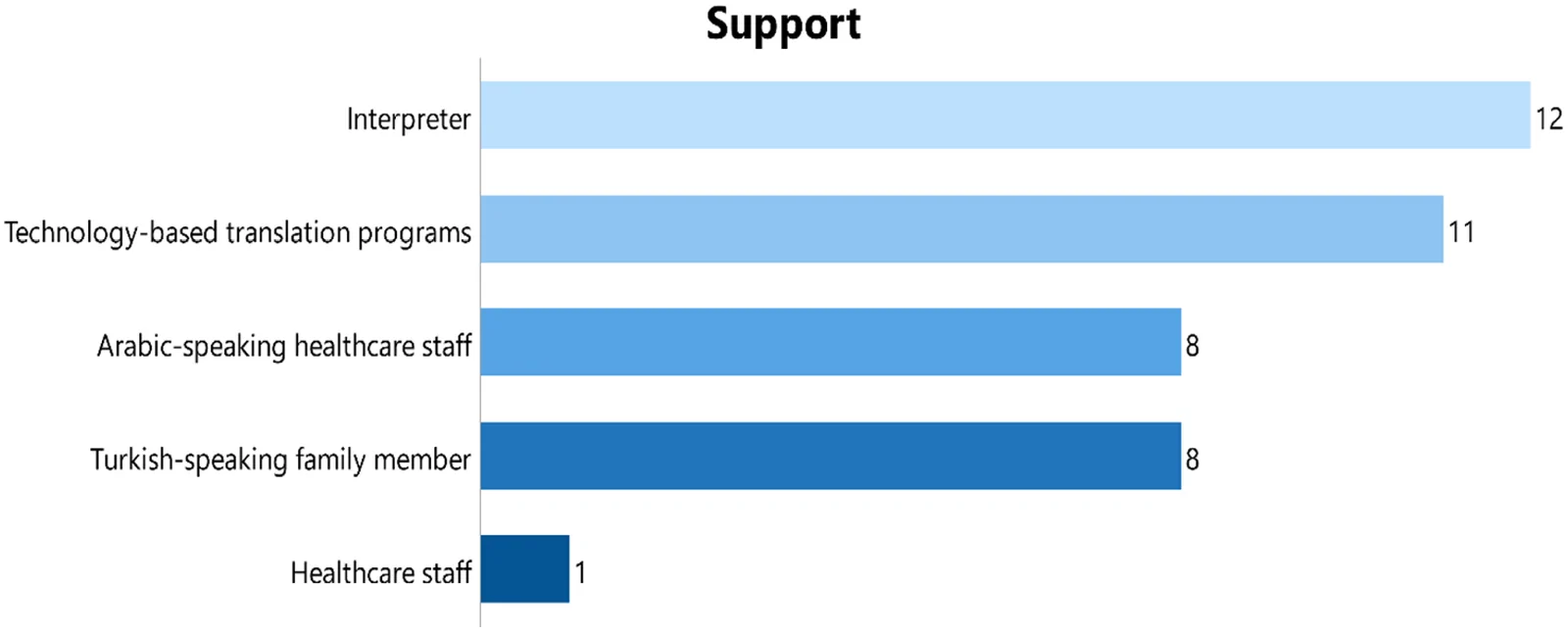
Codes and frequencies related to the “Support” theme.

Figure 6 presents the codes and frequencies for the “Support” theme.

Participants were asked, “Which sources of support are useful to you?” Most nurses said that interpreters were the most helpful resource. They used interpreters when one was available. Many nurses also used mobile translation applications. They viewed these tools as a quick way to address communication problems. They used them more often with younger patients. Staff who spoke Arabic also helped. Their language skills made communication faster and care more culturally appropriate. Family members who spoke Turkish often acted as a bridge between staff and patients. One nurse also asked hospital staff for help when patients requested care from a staff member of the same gender.

P10: *“I rely on technology.” When there’s something they don’t understand, I use translation programs. This is insufficient, but it can be tolerated. If there are interpreters or other friends who know Arabic, I also get support from them.”*

P8: *“…Sometimes we get support from translators, sometimes from healthcare workers who speak Arabic.” We also use technological support resources depending on whether the patients are elderly or young...”*

P5: *“…It varies depending on the situation, for example, if they don’t speak the language, we call a hospital interpreter, or a colleague who knows Arabic helps us. For instance, there is something the patient shouldn’t eat, but they find it culturally acceptable...”*

## 4. Discussion

The findings were discussed in relation to previous research.

### 4.1. Challenges of working with refugees

Healthcare delivery is complex because it involves direct and continuous contact with patients.^[31]^ Differences in language, culture, and daily life can make this work more difficult.^[32]^ Nurses provide care at all hours and face many challenges.^[33]^ Previous studies also show that cultural differences can cause stress for staff caring for refugees. Some studies focus on cross-cultural care.^[34–38]^ Others focus on health beliefs and traditional health practices. Limited skill in the host language can also threaten safe care.^[22,34,39]^ In some regions, geographic closeness and shared language can make care easier.^[11,33,40]^ Nurses in this study also faced cultural challenges. These challenges have continued for more than ten years. They are now a health system issue rather than a short-term crisis. Hatay has a large refugee population. Contact between refugees and staff is part of daily care. Staff must manage language barriers and strong social norms. These norms concern gender, family roles, and health-seeking behavior. The findings, therefore, show a gap in how the system has adapted. They do not reflect only personal conflict. Nurses who spoke Arabic reported fewer problems. This suggests that many barriers can be reduced. Language is not only a personal skill. It is also a key part of safe care. Hospitals should hire bilingual staff and provide trained interpreters. They should also plan staffing for areas with many refugees. Language skills should be treated as a workforce asset. Hospitals in Hatay could offer incentives to bilingual staff. They could also give them priority in hiring. This may reduce strain on public hospitals. It may also move care from crisis response to stable daily practice. Care should not depend on a bilingual worker being present by chance. Better language skills can improve communication and care quality. They can also reduce health inequalities linked to cultural differences. Nursing programs could include medical Arabic training. Hospitals should expand trained interpreter services. Ongoing staff training should also cover cultural competence. Without these steps, nurses must carry most of the burden. This may increase burnout and reduce the quality of patient care.

The findings also show a heavy emotional burden among nurses. They must bridge language gaps while caring for patients with trauma and economic hardship. This can increase fatigue and burnout. Compassion fatigue may be greater in Hatay because the patient load is high and many needs are urgent. Nurses manage this stress each day. Previous refugee studies report the same pattern. Emotional strain is therefore a system issue, not only a personal one. Hospitals should provide mental health support, peer debriefing, and training in stress management. Without this support, nurses may not remain in these roles. Supporting nurses’ mental health is as important as providing language support. Both are needed for safe refugee care.^[33,41,42]^

### 4.2. Difficulties in understanding healthcare needs

Understanding refugees’ health needs supports timely and safe care.^[43]^ It also helps make services fair and sustainable for this vulnerable group.^[34]^ In Türkiye, some health needs may remain hidden. Language and cultural differences can prevent nurses from fully understanding a patient’s symptoms. This can reduce fairness in care. Access to a hospital is not enough. Staff must also understand and value the patient’s account. Nurses need this information when planning treatment and care. Clear assessment of needs supports whole-person care and may reduce barriers to access.^[35,44]^ Some studies identify cultural differences as the main barrier. Others focus on differences in health beliefs and expectations of care.^[12,33,37,45]^ This study found that shame and hidden pain were also important. Some patients viewed discussion of illness as weakness or a loss of privacy. Nurses then had to rely more on visible signs than on patient reports. This may delay diagnosis or treatment. The risk may be greater in reproductive health and after surgery.

Many studies report language barriers when health needs are assessed. Some describe them as a practical communication problem. Others show risks to safe and accurate treatment.^[21,22,38,40,45–47]^ In this study, nurses who spoke Arabic or English understood patient needs more quickly. They could also respond sooner. A shared language is therefore more than a helpful tool. It can affect the quality and safety of care.

Akman et al. (2026) found that cultural competence was linked to more positive caring behaviors among nurses in Istanbul. They also found an unexpected negative link between empathy and cultural competence.^[48]^ The present findings may help explain this result. Nurses with high empathy may notice their own language and cultural limits more clearly. This awareness may lower how they rate their own competence. Quantitative measures may not capture this process. This study adds insight into the feelings and thought processes behind the link.

English education and staff who speak English or Arabic may improve care. However, language skills alone are not enough. Nurses also need training in cultural humility. They must consider migration trauma, religious beliefs, and traditional health practices. Hospitals should support cultural mediators as well as interpreters. These mediators can connect the needs of the Turkish health system with the social and cultural expectations of refugees.

### 4.3. Barriers to accessing healthcare services

Health care can be costly and difficult to use.^[45]^ Refugees may face many barriers when seeking care.^[49]^ Challenges faced by healthcare staff can also limit access and use.^[44]^

Previous studies show that cultural differences can limit access to care. Some focus on different expectations and communication styles. Others show how gender preferences and family roles can conflict with hospital rules.^[22,47,50]^ Refugees and local residents use the same busy system in Hatay. Cultural barriers, therefore, go beyond personal conflict. They may delay hospital visits and increase requests for staff of the same gender. Families may also take a larger role in decisions. These factors can make triage more difficult. They can increase waiting times and staff workload. Cultural differences can therefore place pressure on the whole system. Culturally sensitive care may build trust and improve follow-up. It may also make clinical visits more efficient.

Cultural sensitivity may improve access to care. Language barriers also cause more than communication problems. They can reduce health literacy and make the system hard to use. They can also interrupt follow-up care.^[22,34,38–43]^ Refugees may not understand appointments, referrals, or discharge advice. Their care may then become fragmented. They may also return to emergency units. Limited knowledge of the system can lead to poor use of services. This adds further strain to the health system.

Previous studies also show structural barriers. These include poor guidance, inadequate signs, low health literacy, delays in care, and economic barriers.^[43,46,47]^ These barriers may act together. Poor signs matter more when language support is absent. Economic barriers can also delay care and increase emergency use.

Action is needed at several levels. Hospitals in areas with many refugees should use signs in more than 1 language. They should offer patient navigation support. They should also plan gender-sensitive staffing when needed. Community-based primary care should teach refugees how to use the health system. This may reduce avoidable emergency visits and improve follow-up. Without a system change, nurses will continue to manage these gaps.

### 4.4. Support and successes

Healthcare staff use both institutional and personal support when caring for refugees, who are a vulnerable group. In this study, nurses used interpreters to reduce language and cultural barriers.^[11,38,51,52]^ Previous studies describe trained interpreters as a formal solution. Other studies show that digital tools can provide quick communication support.^[53–57]^

The findings confirm the use of interpreters and mobile translation applications. However, translation applications often serve as emergency support rather than formal clinical tools. They can provide quick help, but they may miss culture, context, and emotion. Use of these informal tools shows a gap in language services. Nurses then fill this gap to protect safe and continuous care.

Support should be part of routine care rather than an informal response. Safe refugee care requires trained medical interpreters. It also requires clear rules for gender-sensitive care. Cultural mediation should be recognized as a nursing skill. Without formal support, individual nurses carry too much of the burden.

Nurses also reported short-term success when relatives acted as interpreters. Coworkers who spoke Arabic also helped. These methods may improve communication, but they create risks. Family interpretation may affect privacy, informed consent, and accuracy. Dependence on bilingual coworkers also creates hidden extra work. This may cause fatigue and an unfair division of tasks. These methods show the nurses’ resilience and skill. They also reveal gaps in the system. Formal, safe, and ethical support is still needed.

### 4.5. Conceptual and theoretical implications

The findings have implications for cultural competence and nursing practice in areas with high migration. Cultural competence is often viewed as an individual nurse’s knowledge and skill.^[58,59]^ The data support a broader system model. The themes show that language services can support or limit good care. These services include trained interpreters and bilingual staff. Recent nursing studies report similar language and cultural barriers.^[10,60,61]^ Nurses also develop informal strategies to keep care focused on the patient. Nursing models for refugee care should include cultural mediation as a core skill.

Fair and whole-person care requires more than staff training. Care models should also include hospital readiness. This includes language support and gender-sensitive staffing. Previous studies describe cultural barriers and the need for training. This study adds a system model of cultural competence. In this model, good care depends on both professional ethics and hospital capacity. The focus moves from the skills of 1 nurse to the conditions that support or limit good care.

## 5. Limitations

This study has several limitations. First, gender was an important theme, but the experiences of male and female nurses were not compared. Such a comparison may have shown gender-specific challenges and coping strategies. Second, the study included only nurses and did not include the views of Syrian refugees. Their views could provide a fuller picture of the care experience. Third, social desirability bias may have affected the interviews. Participants may have emphasized success or limited discussion of problems to meet professional expectations. Finally, the findings are closely linked to Hatay’s social, cultural, and geographic setting. They may not apply directly to other refugee groups or host countries with different health systems and migration policies. Despite these limits, the study provides a detailed account of nursing challenges in an area with a high refugee population.

## 6. Conclusion and recommendations

This study examined the challenges and successful strategies of nurses caring for Syrian refugees in a busy border province. Nurses showed resilience and developed informal coping strategies. However, they worked within major language, cultural, and system barriers. These challenges are not a fixed part of refugee care. Changes in the health system can prevent or reduce many of them. Language skills and cultural mediation are not only personal skills. They are also key parts of safe refugee care. Nurses also face emotional strain and a risk of compassion fatigue. Sustainable refugee care requires mental health and institutional support for nurses. Health systems should replace informal coping methods with reliable language services. They should also provide culturally sensitive care.

### 6.1. Practical impact and institutional response

The findings led to changes in the study hospital. Hospital leaders received the results and took several steps. The hospital hired permanent, trained medical interpreters. They now provide language support across all clinical units. The hospital also opened a Refugee Information and Support Desk. A multidisciplinary team manages the desk. It gives staff guidance on language and cultural issues. These changes made refugee care more efficient. They also reduced hidden work and emotional strain among nurses. This shows that qualitative nursing research can guide change in health systems.

### 6.2. Recommendations for practice and policy

Based on the findings, we propose the following practical recommendations:

#### 6.2.1. Staffing and resources

Hospitals in areas with many refugees should set a minimum interpreter-to-patient ratio. For example, at least 1 trained medical interpreter could serve every 50 refugee admissions. Hiring policies should also favor bilingual nurses. Hospitals may offer financial or professional incentives to nurses who speak Arabic.

#### 6.2.2. Staff training

Ongoing training should go beyond general cultural awareness. It should cover trauma-informed care and gender-sensitive communication. It should also address the safe and ethical use of informal interpreters.

#### 6.2.3. Digital tools

Hospitals should use video interpreter services or medical translation platforms. These tools should use tested clinical terms. They should replace general mobile translation applications.

#### 6.2.4. Mental health support

Hospitals should offer structured peer debriefing and private counseling. This support should be designed for staff in high-pressure refugee settings. It may also reduce compassion fatigue.

#### 6.2.5. Primary care

Community clinics should teach refugees how to use health services. This may reduce avoidable emergency visits and improve follow-up care.

## Acknowledgments

The authors would like to thank all the nurses who participated in this study for sharing their experiences and the hospital administration for their support during the data collection process.

## Author contributions

**Data curation:** Bircan Kara, Sercan Kara.

**Formal analysis:** Bircan Kara, Sercan Kara.

**Methodology:** Bircan Kara.

**Supervision:** Bircan Kara.

**Conceptualization:** Sercan Kara.

**Writing – original draft:** Bircan Kara, Sercan Kara.

**Writing – review & editing:** Bircan Kara, Sercan Kara.
